# A phase 1 study of tazarotene in adults with advanced cancer

**DOI:** 10.1038/sj.bjc.6601169

**Published:** 2003-08-26

**Authors:** P H Jones, R D Burnett, I Fainaru, P Nadolny, P Walker, Z Yu, D Tang-Liu, T S Ganesan, D C Talbot, A L Harris, G J S Rustin

**Affiliations:** 1 MRC Cancer Cell Unit and Cancer Research UK Department of Oncology, University of Cambridge, Hutchison/MRC Research Centre, Cambridge CB2 2XZ, UK; 2 Allergan Ltd, High Wycombe, Bucks HP12 3SH, UK; 3 Allergan Inc., Irvine, CA 92713-9534, USA; 4 Allergan SA, Mougins 06251, France; 5 Cancer Research UK Medical Oncology Unit, Churchill Hospital, Oxford OX3 7LJ, UK; 6 Centre for Cancer Treatment, Mount Vernon Hospital, Middlesex HA6 2RN, UK

**Keywords:** retinoid, tazarotenic acid, oral

## Abstract

Tazarotene is an acetylenic retinoid which is metabolised to tazarotenic acid and which binds selectively to the retinoid receptors RAR*β* and RAR*γ*. The safety, toxicity and pharmacokinetics of oral tazarotene were determined over 12 weeks of treatment in 34 patients with advanced cancer. Commonly seen toxicities were mucocutaneous symptoms, musculoskeletal pain and headache. Dose-limiting toxicities were hypercalcaemia, hypertriglyceridaemia and musculoskeletal pain. The maximum tolerated dose of tazarotene in this schedule is 25.2 mg day^−1^. Plasma concentrations of tazarotenic acid were found to peak rapidly within 1–3 h of dosing and thereafter declined quickly. The *C*_max_ and AUC values on day 0, and weeks 2 and 4 were similar indicating no drug accumulation. The dose-normalised *C*_max_ and AUC values at different dose levels and different study days appeared to be similar indicating linear pharmacokinetics. No objective responses were seen, although stable disease was seen in six out of eight evaluable patients receiving the three highest dose levels of tazarotene (16.8, 25.2 or 33.4 mg day^−1^). We conclude that oral tazarotene is well tolerated when administered daily for 12 weeks, has a favourable toxicity profile compared with other retinoids and merits further investigation as an anticancer therapy.

Retinoids regulate cell proliferation, differentiation and apoptosis in development and adult life (Altucci and Gronemeyer, 2001). They also have anticancer activity in preclinical studies, acting to promote differentiation and/or apoptosis in tumour cells and now have an established role in the treatment of several malignancies, including acute promyelocytic leukaemia (APL), cutaneous T-cell lymphoma and neuroblastoma (Altucci and Gronemeyer, 2001; Smith and Anderson, 2001).

Retinoids act via two families of nuclear transcription factors, the retinoid (RAR*α*, *β*, *γ*) and rexinoid (RXR*α*, *β*, *γ*) receptors (Altucci and Gronemeyer, 2001). These receptors act mainly as RAR–RXR heterodimers, which regulate the transcription of downstream target genes after binding to retinoic acid response elements in their promoters (Kastner *et al*, 1997). Gene targeting studies in mice indicate that the six retinoid receptors have distinct functions; this has encouraged the development of synthetic ligands that bind selectively to different retinoid receptors (Chen *et al*, 1996).

Tazarotene (ethyl 6-[2-(4,4-dimethylthiocroman-6-yl)-ethynyl] nicotinate) and tazarotenic acid, the free acid metabolite of tazarotene, belong to a novel class of retinoids called acetylenic retinoids (Chandraratna, 1996). Tazarotene itself does not bind to retinoid receptors, but tazarotenic acid is a retinoid agonist that binds with high affinity to the receptors RAR*β* and RAR*γ* (Chandraratna, 1996). RAR*β* expression is decreased in breast and lung cancer compared with normal tissues suggesting that regulation of RAR*β* expression has a role in malignant progression (Xu *et al*, 1997; Picard *et al*, 1999). *In vitro* studies reveal that expression of RAR*β* correlates with sensitivity to retinoid-induced growth inhibition and apoptosis in tumour cell lines, suggesting that this receptor has a key role in retinoid-mediated antitumour effects (Liu *et al*, 1996).

Preclinical studies have shown that tazarotene inhibits the growth of various human tumour cell lines (leukaemia, myeloma and cervical and breast carcinoma), head and neck squamous cell carcinoma xenografts and human tumour explants (Allergan Clinical Investigator Brochure – Tazarotene (AGN 190168), 1998). Topically applied, tazarotene has been in clinical use for some years and has proven effective in the treatment of psoriasis and acne (Lebwohl *et al*, 1998). Preliminary evidence suggests that it may possess significant activity in basal cell carcinoma (Peris *et al*, 1999).

In view of the evidence suggesting that tazarotene may have anticancer activity, we conducted the study presented here. The primary goals of the study were to determine the safety profile of tazarotene, the maximum tolerated dose (MTD) of oral tazarotene when administered on a continuous daily schedule to patients with advanced cancer and the pharmacokinetic profile of tazarotene in cancer patients. Patients were assessed for evidence of tumour response or palliative benefit. As retinoids have side effects that may only appear with prolonged administration, patients were offered a 12-week course of daily treatment to determine the safety of longer term tazarotene administration.

## PATIENTS AND METHODS

### Patient selection

Patients 18 years of age or older with histologically confirmed cancer refractory to conventional therapy, who had made an adequate recovery from side effects of all prior therapy were candidates for this study. All patients had to have the ability to follow study instructions. Signed informed consent was obtained prior to any study procedures. Eligibility criteria also included the following: ECOG Performance Status 0–2; life expectancy greater than 12 weeks; serum calcium ⩽2.89 mmol 1^−1^; fasting serum triglycerides ⩽5.7 mmol 1^−1^; no known sensitivity to any of the ingredients in the study medication, no prior systemic retinoid therapy, or vitamin A at dosages >15 000 IU or mg day^−1^, during the previous year; no concurrent administration of drugs that affect the 2C8 cytochrome *P*450 system such as carbamazepine; no uncontrolled systemic disease other than cancer; no known human immunodeficiency virus (HIV)-infection; gastrointestinal malabsorption for any reason; and no concurrent participation in another investigational study or participation within 30 days prior to the start of the study. Women with child-bearing potential (women were considered as having child-bearing potential unless they were postmenopausal, lacked a uterus and/or both ovaries, or had undergone bilateral tubal ligation) were excluded from the study.

Patients who fulfilled the above criteria and had completed 12 weeks of treatment without evidence of disease progression or poor compliance to treatment, and with adequate tolerance of tazarotene, were eligible for entry into a follow-up study, subject to the exclusion criteria in the first part of the study and the provision of further signed informed consent.

### Drug administration

Tazarotene was supplied by Allergan as soft gelatin capsules in a liquid triglyceride vehicle containing either 0.7 or 2.1 mg tazarotene. The study drug was taken as a single daily dose with breakfast. Although a standard breakfast was not specified, patients were encouraged to eat at least two slices of buttered toast or bread and drink a glass of whole fat milk, and to continue such a breakfast for the duration of the study. Treatment compliance was assessed by capsule counts of the returned study drug by the pharmacist at each centre. Patients who had taken <75% of the prescribed study treatment over two consecutive visits in either study were considered noncompliant and were withdrawn from the study.

### Maximum tolerated dose

The maximum tolerated dose (MTD) was defined as the highest dose level at which not more than one of a cohort of three to six patients experienced dose-limiting toxicity (DLT) over a period of 12 weeks. Dose-limiting toxicity was defined as any drug-related grade 3 or 4 toxicity, or any unresolved toxicity that caused treatment to be interrupted for more than 2 weeks.

### Dose-escalation schedule

Two dose-escalation schedules were used in the study. There was no within-patient dose escalation in either stage of the study. At each visit, patients were graded for toxicity according to the NCI Common Toxicity Criteria (version 2.0).

Stage 1 of the study involved dose escalation in cohorts of three to six patients. The starting dose was 1.4 mg tazarotene once daily, based on the preliminary results of a safety and pharmacokinetic study in healthy volunteer subjects (Allergan study 190168-015P). At least three patients in this cohort had to be assessable for toxicity over a 12-week period before the next cohort of patients was enrolled. The dose-escalation protocol, based on assessment of toxicity at 12 weeks, was as follows. If there was no evidence of toxicity greater than grade 1 in the first cohort, dosage was to be escalated to 2.1 mg once daily in the second cohort. If any of the first three patients treated at either dose level experienced DLT, an additional three patients were treated at that dose level. An additional dose (2.8 mg) was to be evaluated after at least three patients in the 2.1 mg cohort had been assessed for toxicity over 12 weeks of treatment, provided MTD was not reached at the first two dose levels.

After commencing the study, the results of an additional toxicology study became available. This study suggested that the MTD was likely to be significantly higher than initially projected. The dose-escalation schedule was therefore redesigned. The second stage of the study used an accelerated dose titration schedule involving treatment of cohorts of one to six patients (Simon *et al*, 1997). In the absence of toxicity greater than grade 1 in at least three patients assessed over a 12-week period at the 2.8 mg dose level, dosage was to be escalated by 100% for subsequent single patients at 4-weekly rather than 12-weekly intervals. If a patient experienced grade 2 toxicity, two additional patients were enrolled at that dose level. At least three patients in a cohort must have been assessable for toxicity over a 4-week period once grade 2 toxicity was reached. If one or both of the additional patients experienced grade 2 toxicity, the dose was subsequently escalated by 33–50% in cohorts of three patients.

If one of the three patients entered at a dose level experienced DLT, up to three additional patients were entered at that dose level. If one of the six patients experienced DLT, dose escalation proceeded with a dose increment of 20–33%, in at least three patients per dose level. However, if a second patient in the cohort experienced DLT, then the MTD was deemed to be exceeded. If de-escalation from the DLT dose involved a 50% dose reduction to a previously investigated dose level (e.g. from 8.4 to 4.2 mg), then a 33% dosage reduction was investigated.

### Dose modifications

Dose reduction due to toxicity was permitted in either study if continuation of treatment was considered appropriate for the patient by the investigator. A period off treatment was allowed to enable adequate recovery from all treatment-related toxicities before restarting treatment at the reduced dose. Patients were withdrawn from the study for progressive disease, unacceptable toxicity or if the investigators decided it was not in the patient's interest to continue treatment.

### Pretreatment and follow-up studies

Patient histories, including performance status, concomitant medications, physical examination and laboratory safety variables, were measured at screening, at each treatment visit during each study and at follow-up by the responsible local laboratories at each centre. The initial study consisted of 11 visits scheduled at ⩽2 weeks before baseline (screening), week 0 (baseline/start of treatment), and weeks 1, 2, 3, 4, 6, 8, 10, 12 (during treatment) and 16 (follow-up). Patients in the continuation study had a baseline visit (corresponding to week 16 of the initial study) and 10 scheduled follow-up visits at weeks 4, 8, 12, 16, 29, 24, 28, 32, 36 (during treatment) and 40 (follow-up). Blood for biochemistry analyses was taken after the patients had fasted for 8 h and prior to dosing with the study drug. The following variables were assessed. Haematology: haemoglobin, mean corpuscular volume (MCV), haematocrit, white blood cell count (WBC) with differential (neutrophils, lymphocytes, monocytes, eosinophils, basophils) and platelets. Biochemistry: sodium, potassium, chloride, magnesium, bicarbonate, creatinine, urea, lactate dehydrogenase (LDH), aspartate aminotransferase (AST), alanine aminotransferase (ALT), gamma-glutamyltransferase (*γ*GT), alkaline phosphatase, total bilirubin, albumin, globulin, total protein, calcium, phosphorus, glucose, uric acid, total glycerides, high-density lipoprotein (HDL), low-density lipoprotein (LDL) and total cholesterol. Semiquantitative analysis for urinary protein and glucose was also performed. A chest X-ray and strict lateral X-ray films of the thoracic, cervical and lumbar spine, and ankles were taken at screening, week 12 during the initial study and at 12-weekly intervals during the continuation study to monitor possible soft-tissue calcification and bone toxicity.

Objective response was evaluated on the basis of tumour measurements made by chest X-ray and other appropriate clinical investigations (skeletal X-ray, isotopic bone or liver scan, computer-assisted tomography scan, magnetic resonance imaging scan or abdominal ultrasound). Established tumour markers that were accepted as a measure of tumour response were also determined. Clinically measurable or evaluable lesions were measured every 4 weeks. Lesions measurable by scanning investigations were measured every 12 weeks. Response was assessed using the UICC/EORTC criteria.

### Pharmacokinetic analysis

Tazarotene and tazarotenic acid concentrations were measured in plasma samples collected before and 1, 2, 3, 4, 6, 9, 12 and 24 h postdose at baseline and weeks 2 and 4. At week 12, samples were collected predose and 1, 2 and 3 h postdose in the first study and at 12-weekly intervals in all patients who completed at least 12 further weeks on treatment in the continuation study. On each blood sampling day, patients were asked to take their assigned dose of study drug with breakfast. Although a standard breakfast was not specified, patients were encouraged to eat a minimum of two slices of buttered toast or bread and drink a glass of whole fat milk on each occasion. Blood was collected into tubes containing ethylene diamine tetra-acetic acid (EDTA) as anticoagulant, gently inverted to ensure adequate mixing, placed on ice for 5 min and then centrifuged for 10 min at 2000 **g** at 4°C. Plasma samples (minimum of 4 ml) were then transferred into labelled glass scintillation vials and stored at −15°C until shipped on dry ice to Allergan, Irvine, CA, USA, for analysis. Tazarotene and tazarotenic acid plasma concentrations were assayed using a validated liquid chromatography–tandem mass spectrometry method (LC–MS/MS) with a concentration range of 0.1–40 ng ml^−1^ for tazarotene and 0.1–100 ng ml^−1^ for tazarotenic acid. The LC–MS/MS method employed tazarotene-D7 and tazarotenic acid-D7 as internal standards to quantitate tazarotene and tazarotenic acid concentrations in human plasma. The specific precursor–product ion pairs used in MRM analysis were: *m*/*z* 352 → 324 (tazarotene); *m*/*z* 359 → 331 (tazarotene-D7); *m*/*z* 324 → 294 (tazarotenic acid); and *m*/*z* 331 → 298 (tazarotenic acid-D7). The retention times of tazarotenic acid and tazarotene were approximately 1.6 and 2.7 min, respectively. The analysis was performed by Allergan, Inc. (Irvine, CA, USA).

For each patient, the following noncompartmental model pharmacokinetic parameters were calculated whenever possible, from the serial plasma tazarotenic acid concentrations:

*C*_max_: maximal observed plasma concentration
*T*_max_: time corresponding to maximal plasma concentration
*K*_e_: apparent terminal phase rate constant estimated by logarithmic-linear regression on the terminal segment of the plasma drug concentration–time curve.
*T*_1/2_: apparent terminal-phase half-life calculated as 0.693/*K*_e_
AUC_0–24_: area under the plasma drug concentration–time curve from 0 to 24 h postdose, calculated by the linear trapezoidal rule
AUC_0–tlast_: area under the plasma drug concentration–time curve from 0 h to the last quantifiable drug concentration time post the first dose, calculated by the linear trapezoidal rule
AUC_0–inf_: area under the plasma drug concentration–time curve from 0 h to time infinity, calculated using the formula AUC_0–inf_=AUC_0–tlast_+*C*_last_/*K*_e_, where *C*_last_ was the last quantifiable drug concentration post the first dose.

Pharmacokinetic parameters *K*_e_, *T*_1/2_ and AUC_0–24_ were calculated from weeks 2 and 4 tazarotenic acid data. *C*_max_ and *T*_max_ were calculated from day 0, weeks 2, 4, 12, 36 and 48 tazarotenic acid data.

Descriptive statistics included mean, standard deviation (s.d.), minimal value (Min), median, maximal value (Max) and coefficient of variation (CV). Pharmacokinetic data analyses were performed by the Pharmacokinetics and Drug Metabolism Department at Allergan (Irvine, CA, USA).

## RESULTS

### Patient characteristics

A total of 34 patients with refractory solid tumours were treated with tazarotene at eight dose levels. Patient characteristics are shown in Table 1

**Table 1:** Summary of demographic and disease characteristics

**Characteristic**	**All patients (*n*=34)**
Age (years): mean (range)	58.5 (39–72)
Male : female; *n*	20 : 14
Caucasian: *n*	34
Weight (kg): mean (range)	72.4 (48–112)
Site of current cancer: *n*	
Colorectal	6
Ovary	5
Mesothelioma	4
Lung	3
Kidney	3
Cervix	3
Breast	2
Skin	2
Other	6
Prior anticancer treatment: *n*^a^	
Chemotherapy	26
Radiation therapy	21
Surgery	16
Laser treatment	1
Cryotherapy	1

a Patients may have received more than one anticancer treatment.

. All patients received tazarotene for at least 7 days, 31 patients received tazarotene for at least 4 weeks, 19 patients received tazarotene for at least 12 weeks. One patient was on tazarotene for 50 weeks. The doses received by patients and reasons for withdrawal from the study are shown in Table 2

**Table 2:** Summary of trial outcome and tazarotene dose levels (mg day^−1^)

**Patient status**	**Total**	**1.4**	**2.1**	**2.8**	**4.2**	**8.4**	**16.8**	**25.2**	**33.6**
Initial study (day 0–week 12)									
Enrolled	34	6	5	3	2	2	3	7	6
Completed	19	6	3	1	1	0	1	3	4
Withdrawn	15	0	2	2	1	2	2	4	2
Disease progression	9	0	1	2	1	1	1	2	1
Adverse events	4	0	1	0	0	1	1	0	1
Patient's request	1	0	0	0	0	0	0	1	0
Investigator's opinion	1	0	0	0	0	0	0	1	0

Continuation study (week 12 onwards)	5	0	1	0	0	0	1	0	3^a^

a Original dose level allocated at entry to the study: dose reduction to 16.8 mg for two patients took place before starting the continuation study.

. Fifteen patients discontinued from the study before 12 weeks: nine patients were withdrawn due to disease progression, four patients were withdrawn due to adverse events, and one patient each was withdrawn at their own request or the investigator's decision. Five of the 19 patients who completed the first study entered the continuation study. The pharmacist at each study centre counted the number of returned capsules and documented this in records maintained at each centre. All patients were judged to have been compliant with the study medication.

The dose of tazarotene was reduced in four patients during the initial study. One patient had two dose reductions (from 33.6 to 25.2 mg day^−1^ and then 16.8 mg day^−1^); the other three patients had one dose reduction (one patient from 33.6 to 25.2 mg day^−1^, and two from 25.2 to 16.8 mg day^−1^).

### Adverse events

Adverse events were reported for 30 of the 34 patients. The adverse events that occurred in three or more patients are summarised in Table 3

**Table 3:** Symptomatic adverse events and biochemical abnormalities of frequency≥10%, related to tazarotene treatment

	**Dose level (mg day^−1^)**																										
	**1.4**			**2.1**			**2.8**			**4.2**			**8.4**			**16.8**			**25.2**			**33.6**			**Cumulative**		
	**(*n*=6)**			**(*n*=5)**			**(*n*=3)**			**(*n*=2)**			**(*n*=2)**			**(*n*=3)**			**(*n*=7)**			**(*n*=6)**			**(*n*=34)**		
**NCI toxicity grade**	**1**	**2**	**3/4**	**1**	**2**	**3/4**	**1**	**2**	**3/4**	**1**	**2**	**3/4**	**1**	**2**	**3/4**	**1**	**2**	**3/4**	**1**	**2**	**3/4**	**1**	**2**	**3/4**	**1**	**2**	**3/4**
Cheilitis	0	0	0	1	0	0	2	0	0	1	0	0	1	0	0	2	0	0	2	3	0	4	1	0	13	4	0
Asthenia	0	1	0	0	1	1	0	2	0	0	1	0	0	0	0	1	0	1	2	0	1	2	3	0	5	8	3
Dry skin	0	0	0	1	0	0	1	0	0	0	0	0	0	0	0	2	0	0	3	1	0	5	1	0	12	2	0
Anorexia	2	0	0	1	1	0	1	1	0	1	1	0	0	0	0	0	0	0	0	1	0	0	0	0	5	4	0
Pruritus	0	0	0	0	0	0	0	0	0	1	0	0	0	0	0	1	0	0	3	0	0	2	2	0	7	2	0
Nausea	0	0	0	0	0	0	1	0	0	1	1	0	0	0	0	0	1	0	2	0	0	1	1	0	5	3	0
Vomiting	0	0	0	0	1	0	2	0	0	0	1	0	0	0	0	0	1	0	1	0	0	2	0	0	5	3	0
Back pain	0	0	0	0	0	0	0	0	0	0	1	0	0	0	0	0	1	0	1	1	1	0	2	0	1	5	1
Headache	0	0	0	1	0	0	1	0	0	1	0	0	0	0	0	0	0	0	2	0	0	0	2	0	5	2	0
Stiffness in joints	1	0	0	0	0	0	0	0	0	0	0	0	0	0	0	1	0	0	2	1	0	0	2	0	4	3	0
Rhinitis	0	0	0	0	0	0	0	0	0	1	0	0	0	0	0	1	0	0	1	0	0	1	2	0	4	2	0
Myalgia	0	0	0	0	0	0	0	0	0	0	0	0	0	0	0	0	0	0	1	1	0	1	3	0	2	4	0
Mouth ulcer	1	0	0	1	0	0	0	0	0	1	0	0	1	0	0	0	0	0	0	0	0	0	1	0	4	1	0
Blocked ears	0	0	0	0	0	0	0	0	0	0	0	0	0	0	0	1	0	0	1	0	0	1	2	0	3	2	0
Alopecia	0	0	0	0	0	0	0	0	0	0	0	0	0	0	0	0	0	0	2	0	0	3	0	0	5	0	0
Sore nose	0	0	0	0	0	0	0	0	0	0	0	0	0	0	0	1	0	0	0	2	0	1	0	0	2	2	0
Abdominal pain	0	0	0	1	0	0	0	2	0	0	0	0	0	0	0	0	0	0	1	0	0	0	0	0	2	2	0
Biochemical																											
Hypertriglyceridaemia	2	0	0	0	0	0	2	0	0	2	0	0	1	0	0	2	1	0	2	2	0	3	1	**1**^a^	14	4	**1**^a^
Hypercholesterolaemia	0	2	**1**^b^	0	1	0	1	0	0	1	0	0	0	0	0	2	1	0	4	0	0	2	2	0	10	6	**1**^b^
Hypercalcaemia	1	0	0	1	0	0	0	0	0	1	0	0	1	0	0	0	0	0	1	0	0	0	0	1	5	0	1
Number of patients with any toxicity>grade 1 at dose-escalation assessment^c^		2	**1**		2	1		2	0		0	0		0	0		2^d^	0		3	1		4	2			

a NCI grade 4 toxicity is shown in bold.

b NCI grade 4, with grade 2 elevation at baseline.

c Dose escalation was after 12 weeks treatment for 1.4 and 2.1 mg dose levels and 4 weeks treatment for other dose levels.

d One of the two patients with grade 2 toxicity at 16.8 mg had a single 24 h episode of nausea and vomiting at 2 weeks, which, although possibly treatment-related, was not assessed in the dosage-escalation decision.

, which records the worst grade of toxicity experienced by each patient during their time on study. Also listed in Table 3 are the numbers of patients at each dose level who experienced toxicity greater than grade 1 at the time when the decision to increase the dose of the next cohort of patients was made (see dose-escalation schedule, above). For the three lowest dose levels, this was after at least three patients had completed 12 weeks of treatment. For higher dose levels, this was after at least one patient had completed 4 weeks of treatment; during this accelerated dose-escalation phase, the majority of toxicities above grade 1 in severity developed after the decision to dose escalate had been taken. Table 4

**Table 4:** Toxicities at or above grade 2 and their time of onset

**Dose (mg)**	**Patient no.**	**Toxicity**	**Grade**	**Time of onset (weeks)**
1.4	0101	Asthenia	2	4
		Hypercholesterolaemia	2	8
	0103	Hypercholesterolaemia^a^	4	1
	0201	Hypercholesterolaemia	2	8
2.1	0105	Asthenia	2	16
		Vomiting	2	16
		Hypercholesterolaemia	2	1
	0206	Asthenia	3	6
		Anorexia	2	4
2.8	0106	Anorexia	2	4
		Abdominal pain	2	4
	0107	Abdominal pain	2	4
		Asthenia	2	4
	0207	Asthenia	2	8
4.2	0208	Asthenia	2	8
	0108	Anorexia	2	8
		Nausea	2	8
		Vomiting	2	8
		Back pain	2	8
8.4		Nil		
16.8	0111	Nausea^b^	2	2
		Vomiting^b^	2	2
	0210	Asthenia	2	2
		Back pain	2	2
		Hypertriglyceridaemia^a^	2	2
		Hypercholesterolaemia	3	12
25.2	0115	Dry skin	2	4
		Cheilitis	2	4
		Sore nose	2	4
		Stiff joints	2	8
	0116	Cheilitis	2	4
		Back pain	2	4
		Sore nose	2	8
		Hypertriglyceridaemia	2	2
	0117	Anorexia	2	4
		Cheilitis	2	8
	0214	Asthenia	3	8
		Back pain	3	8
		Myalgia	2	12
33.6^c^	0112	Hypercalcaemia	3	4
		Joint stiffness	2	4
	0113	Asthenia	2	4
	0114	Cheilitis	2	2
		Dry skin	2	2
	0211	Headache	2	2
	0212	Myalgia	2	2
	0213	Hypertriglyceridaemia	4	2

a Grade 2 at baseline.

b Single episode, not assessed in the dosage-escalation decision.

c Only grade 2 toxicities occurring in the first 4 weeks are listed; numerous other grade 2 toxicities were seen at this dose level (see Table 3).

lists all toxicities greater than grade 1 and their time of onset, apart from at the highest dose level where only grade 2/3 toxicities occurring in the first 4 weeks are listed. Numerous other grade 2/3 toxicities were seen after 4 weeks at the 33.6 mg day^−1^ dose level, see Table 3. The latency of grade 2 toxicity was markedly shortened at the highest dose level.

The commonest symptomatic adverse events were cutaneous symptoms, cheilitis and dry skin (in 17 and 14 patients, respectively). Pruritus, rhinitis, oral ulceration, blocked ears, nasal soreness and alopecia were also seen, but were not severe (grade 1 or 2). Asthenia occurred in 15 patients and was severe (grade 3) in three patients. Grade 3 asthenia developed after 6–12 weeks of treatment. Headache and musculoskeletal side effects occurred in seven patients, but were grade 1 or 2 in severity apart from one patient who developed grade 3 backache after 8 weeks of treatment.

Retinoids may cause skeletal toxicity after prolonged administration, resulting in ossification of ligamentous insertions (Pittsley and Yoder, 1983). All patients had appropriate radiographs at 12-weekly intervals to screen for this side effect but none was detected.

The commonest biochemical abnormalities were hypertriglyceridaemia and hypercholesterolaemia, occurring at grade 1 or 2 severity in 18 and 16 patients, respectively. Mild hypercalcaemia occurred in five patients. Despite small patient numbers, there was evidence to suggest a higher prevalence of biochemical abnormalities at the higher dose levels. Hypertriglyceridaemia occurred in four out of seven patients on 25.2 mg day^−1^ and five out of six patients on 33.6 mg day^−1^, while hypercholesterolaemia was seen in four out of seven patients on 25.2 mg day^−1^ and four out of six patients on 33.6 mg day^−1^. Severe bio-chemical abnormalities were seen in three patients. One patient on the 1.4 mg day^−1^ dose level, with grade 2 hypercholesterolaemia at baseline, developed grade 4 hypercholesterolaemia within 1 week of commencing treatment. One patient at the highest dose level (33.6 mg day^−1^) developed grade 3 hypercalcaemia after 4 weeks on study and a second patient at this dose level developed grade 4 hypertriglyceridaemia after 2 weeks of treatment.

Over the dose range of 1.4–33.6 mg used in this study, two out of six patients developed DLT on 33.6 mg (hypercalcaemia and hypertriglyceridaemia) and one out of six patients had DLT on 25.2 mg (musculoskeletal pain). Thus, the MTD of tazarotene was 25.2 mg.

Five patients, one each on 4.2, 8.4, 16.8, 25.2 and 33.6 mg, died either during or within 30 days of discontinuation of tazarotene; in each case, the cause of death was not drug related.

### Responses

Tumour response was assessed in 19 of the 34 enrolled patients who received the study drug for at least 12 weeks. All patients had progressive disease prior to study entry. There were no complete or partial tumour responses. Nine patients had stable disease at the 12-week assessment, in nine patients there was disease progression and one patient was unevaluable. There were eight evaluable patients at the highest three dose levels of whom six had stable disease (all four patients on 33.6 mg, one of three patients on 25.2 mg and one patient on 16.8 mg). The tumour types of those patients who had stable disease were squamous carcinoma of the lung, renal cell cancer, squamous cell carcinoma of the cervix, ovarian cancer, nonsmall cell lung cancer and mesothelioma. This contrasts with three of 11 evaluable patients with stable disease at the lower dose levels (melanoma, gastric cancer and renal cell cancer). There is no evidence of clinical response from this study.

### Pharmacokinetics

**Figure 1:**
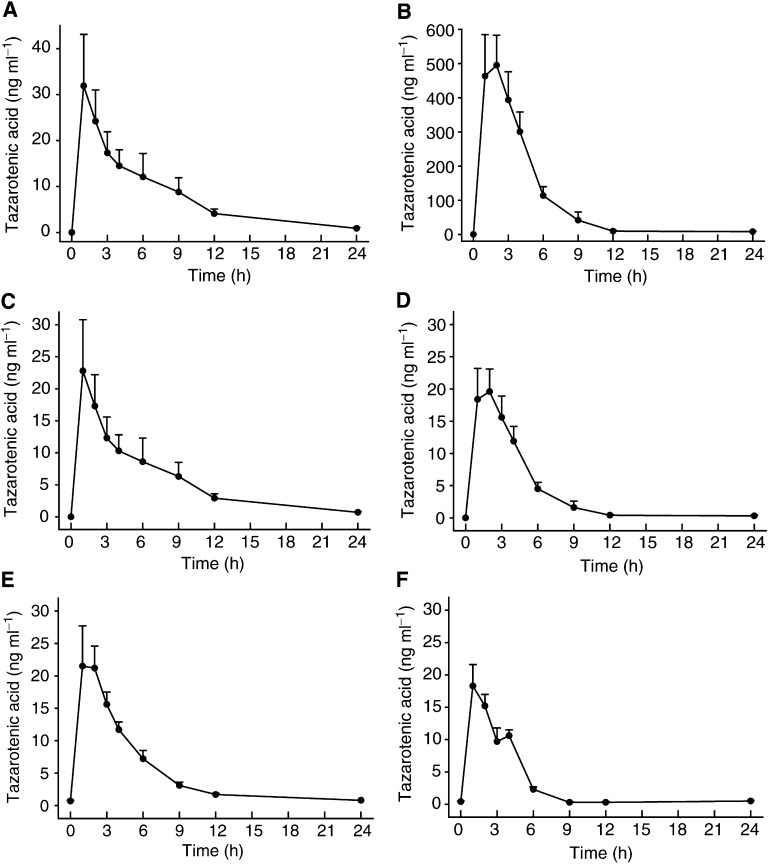
**(A**) Plasma concentrations (mean±s.e.m.) of tazarotenic acid following oral administration of 1.4 mg tazarotene in six cancer patients on day 0. (**B**) Plasma concentrations (mean±s.e.m.) of tazarotenic acid following oral administration of 25.2 mg tazarotene in seven cancer patients on day 0. (**C**) Dose-normalised (1 mg) plasma concentrations (mean±s.e.m.) of tazarotenic acid following oral administration of 1.4 mg tazarotene in six cancer patients on day 0. (**D**) Dose-normalised (1 mg) plasma concentrations (mean±s.e.m.) of tazarotenic acid following oral administration of 25.2 mg tazarotene in seven cancer patients on day 0. (**E**) Dose-normalised (1 mg) plasma concentrations (mean±s.e.m.) of tazarotenic acid following oral administration of 1.4 mg tazarotene in six cancer patients at week 4. (**F**) Dose-normalized (1 mg) plasma concentrations (mean±s.e.m.) of tazarotenic acid following oral administration of 25.2 mg tazarotene in five cancer patients at week 4.

Samples were analysed for concentrations of tazarotene and its active metabolite tazarotenic acid. Plasma tazarotene concentrations were not quantifiable in most (79%, 670 out of 848 samples) samples. The single highest plasma tazarotene concentration throughout the study was 24.0 ng ml^−1^. Peak plasma tazarotenic acid concentration was reached rapidly within 1–3 h of dosing and thereafter declined quickly. Representative concentration–time curves are shown in Figure 1A–F

and a summary of the pharmacokinetic parameters is given in Table 5

**Table 5:** Summary of the pharmacokinetic parameters of tazarotenic acid following single-dose oral administration of Tazarotene on day 0 and at 12 weeks

**Dose taken (mg)**	***C*_max_ day 0 (ng ml^−1^)**	***T*_max_ day 0 (h)**	**AUC_0–inf_ day 0 (ng h ml^−1^)**	***T*_1/2_ day 0 (h)**	***C*_max_** **week 12 (ng ml^−1^)**
1.4	40.8±18.3	3.2±3.5	195±38	5.62 ± 0.86	29.6
(*N*=6)	(20.4, 41.7, 66.3)	(1, 1, 9)	(159, 188, 243)	(4.62, 5.65, 6.78)	(*N*=6)
2.1	68.8±27.8	3.6±3.3	279±64	8.36±3.59	41.9
(*N*=5)	(35.2, 73.2, 107)	(1, 3, 9)	(204, 308, 353)	(5.35, 8.14, 14.3)	(*N*=3)
2.8	70.8±28.1	2.7±1.5	394±60	7.96±2.87	89.4
(*N*=3)	(45.4, 65.9, 101)	(1, 3, 4)	(328, 412, 443)	(4.81, 8.67, 10.4)	(*N*=1)
4.2	72.9±2.1	3.5±3.5	359±1	4.05±0.36	97.8
(*N*=2)	(71.4, 72.9, 74.3)	(1, 3.5, 6)	(358, 359, 360)	(3.79, 4.05, 4.30)	(*N*=1)
8.4	126±96	2.5±0.7	614±3	14.2±9.6	—
(*N*=2)	(57.8, 126, 194)	(2, 2.5, 3)	(612, 614, 616)	(7.44, 14.2, 21.0)	
16.8	377±112	3.0±1.0	1751±358	15.4±14.3	325
(*N*=3)	(306, 318, 506)	(2, 3, 4)	(1341, 1915, 1998)	(1.25, 15.0, 29.9)	(*N*=3)
25.2	561±236	2.1±1.1	2394±841	3.33±2.56	367
(*N*=7)	(191, 559, 859)	(1, 2, 4)	(1540, 2287, 3677)	(1.32, 2.35, 8.53)	(*N*=1)
33.6	903±267	1.5±0.6	3467±707	8.01±6.51	829
(*N*=4)	(680, 823, 1286)	(1, 1.5, 2)	(2809, 3425, 4208)	(1.20, 7.90, 15.1)	(*N*=1)

Pharmacokinetic parameters are presented as mean±s.d. (minimum, median and maximum).

. The mean apparent elimination half-lives on day 0 ranged from 33.3 to 15.4 h (see Table 5). There was no change in the concentration–time curves with repeated dosing (Figure 1A–F). There was no significant difference between *C*_max_ and AUC values on day 0, and weeks 2 and 4, indicating no drug accumulation or self-induction of metabolism (data not shown). The peak tazarotenic acid concentrations at week 12 were also similar to those on day 0 and weeks 2 and 4, although patient numbers were insufficient for statistical analysis (Table 5). Pharmacokinetic data were available for only a single patient following week 12, and although significant serum levels of tazarotenic acid were detected, no conclusions could be drawn from such limited information. The dose-normalised (to 1 mg) *C*_max_ and AUC values at different dose levels and different study days appeared to be similar indicating linear pharmacokinetics. There was no evidence to suggest any differences in the pharmacokinetic profile of tazarotenic acid in cancer patients from that found in other studies in healthy volunteers.

## DISCUSSION

A key requirement for candidate synthetic retinoids for use in cancer treatment is an acceptable toxicity profile that enables prolonged administration either as a single agent or in combination with other agents such as cytotoxics or interferon (Lippman *et al*, 1992; Khuri *et al*, 2001). This study shows that tazarotene is well tolerated up to the MTD of 25.2 mg day^−1^ given daily for 12 weeks.

Although the range of adverse events seen in this study is similar to that reported for other retinoids, the frequency of some symptomatic adverse events at the MTD was lower with tazarotene. The commonest side effects were mucocutaneous (cheilitis and dry skin), which were experienced by the majority of patients at the two highest dosages investigated, but which were never of greater than grade 2 severity. Similar toxicity has been reported with 9-*cis* retinoic acid (9-Cis RA) and with all *trans* retinoic acid (ATRA) (Lee *et al*, 1993; Miller *et al*, 1996; Park *et al*, 2000). Despite the small numbers of patients in this study, it seems that there is a lower frequency of severe mucocutaneous toxicity with tazarotene than with ATRA for which mucocutaneous toxicity was a DLT in one study (Park *et al*, 2000).

There were some differences in the adverse events in this study compared with other retinoids. Ocular toxicity (dry eyes and conjunctivitis), which occurs in 25–40% of patients treated with 13-*cis* retinoic acid (13-Cis RA) or ATRA, was only seen in one patient in this study (Lee *et al*, 1993; Miller *et al*, 1996). Likewise, headache, which is frequent and may be severe and dose limiting with 9-Cis RA or ATRA, was reported by only seven or 34 patients and was usually mild in severity (Lee *et al*, 1993; Miller *et al*, 1996). There was no evidence of an increased frequency or greater severity of symptomatic side effects with prolonged dosing. Thus tazarotene has a favourable symptomatic side effect profile compared with 9-Cis RA, 13-Cis RA and ATRA. The improved tolerance of tazarotene may be due to its selective activity on RAR*β* and RAR*γ* receptors, in contrast to ATRA which is a pan RAR agonist and 9-Cis RA which is a pan RAR and RXR agonist (Altucci and Gronemeyer, 2001).

Hypertriglyceridaemia and hypercholesterolaemia are common toxicities with retinoids, and with tazarotene proved to be dose limiting (Bershad *et al*, 1985; Lee *et al*, 1993; Miller *et al*, 1996). Hypercalcaemia was also encountered in six patients, but there was no evidence of ectopic ossification in this study. This side effect is associated with prolonged (greater than 21 months) administration of 13-Cis RA and 9-Cis RA (Pittsley and Yoder, 1983; Lawrence *et al*, 2001).

Tazarotene has ideal pharmacokinetic properties for long-term administration. Plasma concentrations of tazarotenic acid displayed linear pharmacokinetics and there was no evidence of variation in pharmacokinetics with prolonged oral dosing. This is in contrast to ATRA where a continuous oral administration is associated with a progressive decline in plasma concentrations with time, so that the AUC is only 20% of the day 1 value by day 28 (Muindi *et al*, 1992; Smith *et al*, 1992). This effect appears clinically significant and is associated with retinoid resistance and relapse in patients with APL. A similar decline in drug concentration with time occurs with prolonged dosing with 9-Cis RA above a threshold of 140 mg^2^ day^−1^ (Miller *et al*, 1996). The stable pharmacokinetic profile of tazarotene is a major advantage in cancer patients, where schedules often involve prolonged administration. In addition, there is a high level of consistency in plasma tazarotenic acid concentrations between patients treated at the same and different dose levels, suggesting rapid and predictable absorption of tazarotene in the present formulation.

In conclusion, tazarotene is a selective activator of RAR*β* and RAR*γ*. Given orally over 12 weeks, it is well tolerated in cancer patients and an ideal agent for further investigation in cancer patients, either as a single agent or in combination with other drugs, using a dose of 25.2 mg day^−1^.
